# Improving Hereditary Hemorrhagic Telangiectasia Molecular Diagnosis: A Referral Center Experience

**DOI:** 10.3390/genes14030772

**Published:** 2023-03-22

**Authors:** Cinthia Aguilera, Ariadna Padró-Miquel, Anna Esteve-Garcia, Pau Cerdà, Raquel Torres-Iglesias, Núria Llecha, Antoni Riera-Mestre

**Affiliations:** 1 Hereditary Hemorrhagic Telangiectasia Unit, Hospital Universitari de Bellvitge, Institut d’Investigació Biomèdica de Bellvitge (IDIBELL), 08907 L’Hospitalet de Llobregat, Spain; caguilerar@idibell.cat (C.A.); apadro@bellvitgehospital.cat (A.P.-M.); aesteveg@bellvitgehospital.cat (A.E.-G.); pcerda@idibell.cat (P.C.); rtorresi@bellvitgehospital.cat (R.T.-I.); 2 Genetics Laboratory, Laboratori Clínic Territorial Metropolitana Sud, Hospital Universitari de Bellvitge, Institut d’Investigació Biomèdica de Bellvitge (IDIBELL), 08907 L’Hospitalet de Llobregat, Spain; nllecha@bellvitgehospital.cat; 3 Clinical Genetics Unit, Laboratori Clínic Territorial Metropolitana Sud, Hospital Universitari de Bellvitge, Institut d’Investigació Biomèdica de Bellvitge (IDIBELL), 08907 L’Hospitalet de Llobregat, Spain; 4 Internal Medicine Department, Hospital Universitari de Bellvitge, Institut d’Investigació Biomèdica de Bellvitge (IDIBELL), 08907 L’Hospitalet de Llobregat, Spain; 5 Faculty of Medicine and Health Sciences, Universitat de Barcelona, 08907 L’Hospitalet de Llobregat, Spain

**Keywords:** hereditary hemorrhagic telangiectasia, genetic testing, variant of unknown significance, RNA, splicing

## Abstract

Background: Hereditary hemorrhagic telangiectasia (HHT) is a rare vascular disease inherited in an autosomal dominant manner. Disease-causing variants in endoglin (*ENG*) and activin A receptor type II-like 1 (*ACVRL1*) genes are detected in more than 90% of the patients undergoing molecular testing. The identification of variants of unknown significance is often seen as a challenge in clinical practice that makes family screening and genetic counseling difficult. Here, we show that the implementation of cDNA analysis to assess the effect of splice site variants on mRNA splicing is a powerful tool. Methods: Gene panel sequencing of genes associated with HHT and other arteriovenous malformation-related syndromes was performed. To evaluate the effect of the splice site variants, cDNA analysis of *ENG* and *ACVRL1* genes was carried out. Results: three novel splice site variants were identified in *ENG* (c.68-2A > T and c.1311+4_1311+8del) and *ACVLR1* (c.526-6C > G) genes correspondingly in three individuals with HHT that met ≥ 3 Curaçao criteria. All three variants led to an aberrant splicing inducing exon skipping (*ENG*:c.68-2A > T and *ACVRL1*:c.526-6C > G) or intron retention (*ENG*:c.1311+4_1311+8del) allowing the confirmation of the predicted effect on splicing and the reclassification from unknown significance to pathogenic/likely pathogenic of two of them. Conclusions: RNA analysis should be performed to assess and/or confirm the impact of variants on splicing. The molecular diagnosis of HHT patients is crucial to allow family screening and accurate genetic counseling. A multidisciplinary approach including clinicians and geneticists is crucial when dealing with patients with rare diseases.

## 1. Introduction

Hereditary hemorrhagic telangiectasia (HHT), also known as Rendu-Osler-Weber syndrome (ORPHA774), is a rare autosomal dominant multisystemic vascular disease characterized by telangiectasia and larger vascular malformations (VMs), with a prevalence of 1 in 6000 [1,2,3,4]. Although more than 800 pathogenic variants in more than five genes have been reported to be associated with HHT, variants in endoglin (*ENG*; located on chromosome 9q34.11) and activin A receptor type II-like 1 (*ACVRL1*; located on chromosome 12q13.13) genes are present in more than 90% of the patients that undergo molecular testing. They cause HHT1 (OMIM# 187300) and HHT2 (OMIM# 600376), respectively [5,6,7]. Pathogenic/likely pathogenic variants in *SMAD4* (encoding the transcription factor Smad4) have been described in less than 2% of the HHT population associated with juvenile polyposis/HHT overlap syndrome [8]. Endoglin (encoded by *ENG* gene) is an auxiliary co-receptor at the endothelial cell surface that promotes BMP9 signaling through the activin receptor-like kinase 1 (also known as ALK1; encoded by the *ACVRL1* gene). Both proteins contribute to the signaling hub formed by BMP9-endoglin-ALK1-Smad with a high impact on angiogenesis [9,10,11].

HHT can be diagnosed through either molecular genetic testing or using the Curaçao clinical criteria (recurrent epistaxis, cutaneous/mucosal telangiectasia, visceral involvement, and a first-degree relative with HHT). However, HHT is a disease with an age-dependent penetrance. Approximately 90% of the patients show symptoms by the fifth decade of age, but clinical onset is highly variable, ranging from early childhood to late adulthood. This is the reason why genetic testing is a useful tool in diagnosing young patients [1,5,7,12]. Telangiectasia is a classic HHT-associated vascular lesion caused by dilated postcapillary venules directly connected with dilated arterioles losing the capillary bed [5,13]. In particular, telangiectasia situated in the nasal mucosa causes spontaneous and recurrent epistaxis, which is the most common and usually the earliest clinical manifestation of HHT [1,5,14]. Rare diseases are known to face several challenges, such as late diagnosis and misdiagnosis, lack of effective therapies, and proper follow-up tools [15]. Both epistaxis and telangiectasia may be perceived as mild, unrelated, common symptoms delaying HHT diagnosis. Referral HHT Units are crucial for early diagnosis, appropriate management, and the identification of at-risk, asymptomatic individuals.

Although endoglin and ALK1 are components of the same BMP9 receptor complex, they are structurally and functionally different proteins. Thus, pathogenic variants in their genes are related to different clinical phenotypes [7,11]. Pulmonary and brain arteriovenous malformations (AVMs) are more common in patients with HHT1, while vascular hepatic malformations are more frequent in HHT2 [5,12,16]. Of note, there is a high level of interfamilial and intrafamilial phenotypic variability in the vascular involvement and clinical manifestations [5,17]. Therefore, genetic testing is essential for diagnostic confirmation and family screening [5,7,18].

According to the scientific literature and HHT databases, all different types of genetic variants have been reported in *ENG* and *ACVRL1* genes; however, missense variants account for more than half of the variants detected in these genes. Interestingly, only 7.5% (43 out of 572) and 15% (79 out of 510) of the pathogenic or likely pathogenic variants currently reported in *ACVRL1* and *ENG*, respectively, are splice site variants [19,20]. Variants that affect the sequence of canonical acceptor (CAG/GUAAGU) and donor (NYAG/G) splice sites may alter the process of intron removal, frequently leading to single exon skipping. Nonetheless, if the splice site is weak and a variant uncovers a cryptic splice site, it could lead to the inclusion of an intron fragment into the mature RNA [21,22]. Canonical splice site variants located in haploinsufficient genes are usually classified as pathogenic, whereas variants affecting non-canonical splice site regions tend to be classified as variants of unknown significance and cDNA/RNA analysis or in vitro assays are crucial to reclassify these variants [23,24].

Here, we describe three novel splice site variants identified in the *ACVRL1* (c.526-6C > G) and *ENG* (c.1311+4_1311+8del and c.68-2A > T) genes in three individuals with HHT. In order to functionally verify the effect of these variants on the splicing process, cDNA analysis was performed. 

## 2. Materials and Methods

### 2.1. Subjects of Study

The three patients were followed up at the reference HHT Unit in Bellvitge University Hospital which provides medical care for adult patients with HHT from all over Catalonia (Spain). From 2011 to December 2022, 451 patients with a definite clinical diagnosis of HHT (meeting ≥ 3 Curaçao criteria) and/or a positive genetic test have attended the unit. The routine HHT screening is explained elsewhere [9,25].

All three patients provided written informed consent to enroll in this study following our Clinical Research Ethics Committee requirements. Personal and clinical data collected for the study are in line with the Spanish Data Protection Act (Ley Orgánica 3/2018 de 5 de diciembre de Protección de Datos Personales). The study was approved by the Clinical Research Ethics Committee of the Hospital Universitari de Bellvitge (approval number PR203/21). 

Patient 1 is a 65-year-old female with a personal medical history of atrial fibrillation and heart failure, with severe mitral and tricuspid insufficiency requiring valvular repair procedures. Anticoagulant therapy was contraindicated, and a left atrial appendage occlusion was performed in 2020. She was diagnosed with HHT as she met all four Curaçao criteria (Table 1). On physical examination, fingertips and lips muco-cutaneous telangiectasias were observed. She reported recurrent epistaxis since she was eight years old and she had recently developed severe anemia that was disproportionate to the severity of epistaxis. This led to a complete gastrointestinal (GI) evaluation that showed multiple telangiectasia. She required multiple weekly red blood cell transfusions and diverse argon plasma coagulation sessions. Treatment with intramuscular long-acting release octreotide was started, but no improvement in hemoglobin levels was observed after six months. A switch to intravenous bevacizumab (5 mg/kg) was performed, and anemia was resolved, with a maintenance dose of bevacizumab every five weeks. No pulmonary involvement was observed, but an abdominal computed tomography (CT) showed multiple diffuse hepatic telangiectasia with arterioportal and portovenous shunts, requiring a hospital admission in March 2022 due to a hepatic encephalopathy. The patient reported a positive family history on her maternal side, with two out of her three siblings affected (Figure S1a).

Patient 2 is a 73-year-old female with a medical history of recurrent deep venous thrombosis in her right lower limb under extended anticoagulant treatment. She was diagnosed with HHT after meeting three out of four Curaçao criteria (Table 1). The patient reported severe episodes of recurrent epistaxis since childhood, which exacerbated after anticoagulation treatment was initiated. She developed iron-deficiency anemia that resolved with oral iron supplementation. On clinical examination, multiple telangiectasias on the fingertips and inside the oral cavity were detected. A thoracic CT scan showed a pulmonary arteriovenous (AV) fistula in the left lower lobe with a normal abdominal CT scan. Two of her maternal aunts and one out of her three siblings were also affected (Figure S1b). 

Patient 3 is a 53-year-old female with a medical history of celiac disease who was diagnosed with HHT after meeting all Curaçao criteria (Table 1). GI telangiectasias were identified during celiac disease follow-up. She suffered from mild recurrent epistaxis and presented multiple telangiectasias in her fingertips. She has never had anemia but is receiving oral iron due to an iron deficiency. She had a Barzilai classification grade I at transthoracic contrast echocardiography, therefore no pulmonary AV fistulae were expected at CT [26]. An abdominal CT showed hepatic VMs. She reported a family history through her paternal side, with an uncle and two of her cousins affected (Figure S1c). 

### 2.2. Gene Sequencing Panel

DNA was extracted from peripheral blood samples using an automated extraction method (Maxwell RSC Whole Blood DNA Kit, Promega, Madison, WI, USA) and following the manufacturer’s instructions.

A targeted gene sequencing panel was designed to evaluate the reported genes associated with HHT and other arteriovenous malformation related syndromes (*ACVRL1*, *BMPR2*, *BMP10*, *BMPR1A*, *ELMO2*, *ENG*, *EPHB4*, *GDF2*, *PIK3CA*, *RASA1*, *SMAD1*, *SMAD4*, *SMAD6*, *TEK*). Nonacus Cell3™ Target Enrichment System (protocol v1.2.2, Nonacus, Birmingham, UK) was used. This method is based on the enzymatic fragmentation of 100 ng of genomic DNA followed by end-repair and A-tailing. Adapter sequences for massive parallel sequencing are then joined to the DNA fragments. In addition, unique molecular indexes (UMIs) are added to identify individual samples and allow sample multiplexing. All the DNA fragments from selected individuals are then mixed to create the sequencing library. The fragmented genomic DNA library is hybridized to the biotinylated capture customized probes. Subsequently, streptavidin beads are used to recover those DNA fragments that are attached to the biotin probes. The library generated was sequenced with a MiSeq sequencer (Illumina, San Diego, CA, USA), following the protocol developed by the company, and generating 2 × 150 pair-end reads. 

### 2.3. Panel Analysis

Bioinformatic analysis was carried out using the Datagenomics platform version 1.4.0 (Health in Code, Valencia, Spain). High-quality reads were aligned to the human reference genome (GRCh37/hg19), and potential duplicate paired-end reads were removed. Reads with a mapping quality <20 and bases with quality <30 were discarded. Copy number analysis was performed using VarSeq software v.2 (Golden Helix, Inc, Bozeman, MT, USA). Variants were filtered for allele frequencies ≤ 1/100 in the gnomAD v.2 database [27] and their predicted impact on the protein (nonsense, frameshift, splice site, and missense). Splice site variants were evaluated using Splice AI [28] and Varseak [29] (developed by JSI Medical Systems GmbH, Ettenheim, Germany) tools. Variants were classified following the American College of Medical Genetics and Genomics and the Association for Molecular Pathology (ACMG/AMP) guidelines [30].

### 2.4. RNA Analysis

RNA was extracted from fresh whole blood samples using the Maxwell^®^ RSC simply RNA Blood Kit (Promega, San Diego, CA, USA) according to the manufacturer’s protocol. cDNA synthesis was carried out using the PrimeScriptTM RT reagent Kit (Takara Bio Inc., Kusatsu, Japan). Primers amplifying the region, including exons 1 to 5 and 8 to 12 of the *ENG* gene (NM_001114753.3) and exons 4 to 7 of the *ACVRL1* gene (NM_000020.3) were designed in order to analyze the effect on mRNA splicing. The sequences of all primers are available in Table S1. Each amplicon was amplified by PCR at standard conditions and purified using ExoSAP-IT (Applied Biosystems, Foster City, CA, USA) following the manufacturer’s instructions. Then, the purified PCR products were sequenced using the Big Dye Terminator^®^ (V 3.1) Cycle Sequencing Kit (Applied Biosystems). Electropherograms were analyzed using Mutation Surveyor V5.1.2 software (SoftGenetics, LLC, State College, PA, USA). To predict the effect of splice site variants on the protein, the Expasy translation tool was used [31].

## 3. Results

**Figure 1:**
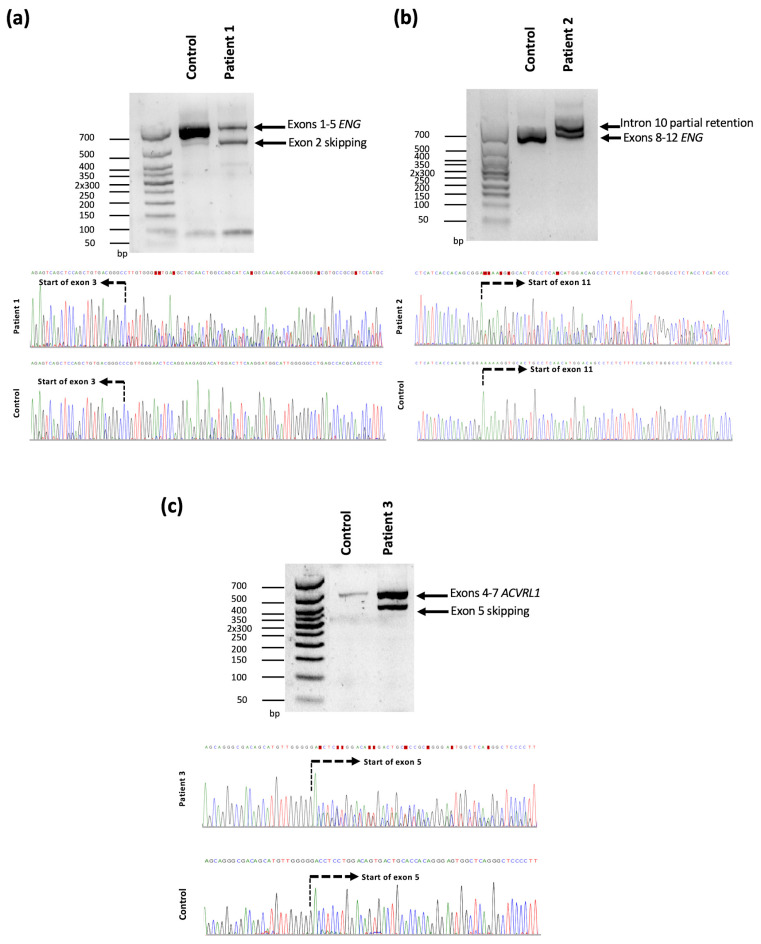
Molecular characterization of the splice site variants identified in *ENG* and *ACVRL1*. (**a**) cDNA analysis of the *ENG* variant NM_001114753.3:c.68-2A > T was identified in patient 1. The PCR amplification products of exons 1 to 5 show two fragments, one of them smaller compared to the control sample. Reverse Sanger sequencing chromatographs indicate the skipping of exon 2. The start of exon 3 is indicated by a dashed line. (**b**) cDNA analysis of the variant NM_001114753.3:c.1311+4_1311+8del in *ENG*. Amplification of exons 8 to 12 result in two bands, one of them bigger than the band present in the control sample. Sanger sequencing chromatographs show a partial intron 10 retention. The start of exon 11 is indicated by a dashed line. (**c**) cDNA analysis of NM_000020.3:c.526-6C > G *ACVRL1* variant. Amplification of exons 4 to 7 of *ACVRL1* show two bands in patient 3, one of the fragments is smaller compared with the control sample. Sanger sequencing revealed the skipping of exon 5. The start of exon 5 is indicated by a dashed line.

**Figure 2:**
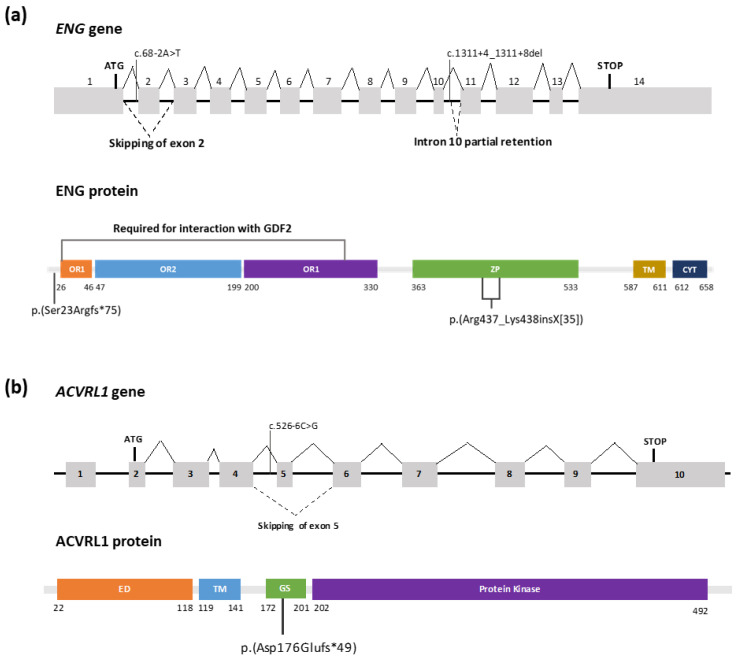
Schematic structure of *ENG* (NM_001114753.3, NP_001108225.1) and *ACVRL1* (NM_000020.3, NP_000011.2) genes illustrating the predicted variant effect on splicing and protein. (**a**) Predicted effect of the variants identified in *ENG* gene. The variant c.68-2A > T would lead to exon 2 skipping and is predicted to introduce a frameshift with a premature stop codon at the protein level. The variant c.1311+4_1311+8del induces a partial intron 10 retentions introducing 35 amino acids in the protein sequence. (**b**) The variant c.526-6C > G identified in the *ACVRL1* gene would lead to exon 5 skipping and the introduction of a frameshift with a premature stop codon at the protein level. Exons are shown as boxes and introns as lines. Protein functional domains are shown as boxes. The variants are indicated with vertical lines and the amino acid insertion is delimited by gray lines. Orphan domain (OR), Zona Pelucida domain (ZP), Transmembrane domain (TM), Cytoplasmic domain (CYT), Extracellular domain (ED), Glycine and Serine rich region (GS).

Gene panel analysis led to the identification of a novel variant, NM_001114753.3:c.68-2A > T, located in the first intron of the *ENG* gene in patient 1. Sanger sequencing confirmed the presence of the variant. Splice AI and Varseak in silico tools indicated a high probability that the variant affected splicing (score 0.99 and class 5, respectively), leading to exon skipping or using a cryptic splice site situated 9 nucleotides downstream 3′. The variant was initially classified as pathogenic according to ACMG/AMP guidelines: (i) it was located on a 3′ canonical acceptor splice site, and the loss of function mechanism is known to cause the disease (PVS1-very strong); (ii) it was absent from controls (PM2-supporting); and (iii) the phenotype is highly specific for HHT (PP4-supporting). In order to confirm the impact of the variant on mRNA splicing, cDNA analysis was performed on the patient and a control sample. Amplification of exons 1–5 of the *ENG* gene resulted in a smaller fragment, suggesting exon skipping (Figure 1a). Sanger sequencing results allowed the confirmation of exon 2 skipping, which led to a frameshift with a premature stop codon NP_001108225.1:p.(Ser23Argfs*75) and caused a loss of function (Figure 2a). Due to the location of the premature stop codon, it was predicted that it would activate the nonsense mediated decay (NMD) machinery.

In patient 2, the variant NM_001114753.3:c.1311+4_1311+8del in *ENG* was identified by gene panel analysis and confirmed by Sanger sequencing. The variant located in the 5′ donor splice site had a high probability of affecting splicing according to Splice AI and varSEAK in silico tools (0.9 and class 5, respectively) by using a cryptic splice site located 69 downstream 5′. No similar variants have been previously reported. The variant was classified as a variant of unknown significance according to the following ACMG/AMP criteria: (i) it was absent from controls (PM2-supporting), (ii) it was predicted to have an effect on splicing (PP3-supporting) and (iii) the phenotype is highly specific of HHT (PP4-supporting). To analyze its impact on splicing, cDNA amplification of exons 8 to 12 of the *ENG* genes were carried out. As a result, a bigger fragment could be observed in the patient compared to the control sample, suggesting intron retention. Sanger sequencing analysis showed that 105 nucleotides of the intron 10 sequence were incorporated in the mRNA (Figure 1b). At the protein level, the variant is predicted to introduce 35 amino acids NP_001108225.1:p.(Arg437_Lys438insX[35]) in the zona pelucida (ZP) protein domain that is involved in oligomerization and protein-protein interactions [32] (Figure 2a). In consonance with the results obtained, the variant NM_001114753.3:c.1311+4_1311+8del was reclassified as likely pathogenic according to the ACMG/AMP guidelines (adding PM4-moderate criteria in agreement with the protein length change).

Finally, the variant NM_000020.3:c.526-6C > G in the *ACVRL1* gene was identified in patient 3. Sanger sequencing confirmed the presence of the variant. Splicing prediction tools indicated a probable effect on splicing (Splice AI, score 0.4 and VarSEAK class 5) by using a cryptic splice site located 60 nucleotides downstream 3′. In agreement with the ACMG/AMP guidelines the variant was classified as a variant of unknown significance: (i) it was absent in population databases (PM2-supporting), (ii) it was predicted to affect splicing (PP3-supporting) and (iii) the phenotype is highly specific of HHT (PP4-supporting). cDNA analysis was performed in order to evaluate the effect of the c.526-6C > G variant on splicing. Amplification of exons 4 to 7 of the *ACVRL1* genes resulted in two bands, one of them being a smaller fragment compared to the control sample, suggesting an exon skipping (Figure 1c). Sanger sequencing analysis confirmed the skipping of exon 5 that was predicted to cause a frameshift with premature stop codon NP_000011.2:p.(Asp176Glufs*49) (Figure 2b) that would activate the NMD mechanism and lead to a loss of function. Considering the results obtained, the variant was reclassified as pathogenic in accordance with the ACMG/AMP guidelines (PM2-supporting, PVS1-very strong, and PP4-supporting).

## 4. Discussion

Splicing is a key process in eukaryotic cells and its disruption is one of the main molecular causes of rare genetic diseases [33,34]. Disturbances in the splicing could lead to the introduction of a premature stop codon or variations in the protein structure due to in-frame changes. Canonical splice site variants situated within 2bp of an exon junction are usually considered as loss of function variants in genetic diseases. Nonetheless, variants located at positions 1-5bp of the splicing donor site or even more distally, 24bp of the acceptor splice site could also lead to an aberrant mRNA splicing [35,36]. It has been reported that the identification of non-canonical splice site variants increases the diagnostic yield to at least 35% [22].

The degree and the nature of the disruption required for presenting clinical manifestations could vary depending on the gene or even the exon. Recessive genes tend to be more permissive than haploinsufficient genes to splice site variations. Multiple in silico tools are available for predicting the impact of variants on pre-mRNA splicing. However, functional analysis by RNA (and/or cDNA), protein, or in vitro assays using minigenes are needed to validate and evaluate the consequences of the splicing process [22,30].

Here, we report three novel splice site variants (c.68-2A > T, c.1311+4_1311+8del in *ENG* gene and c.526-6C > G in *ACVRL1* gene) identified in three patients with a clinical diagnosis of HHT and ≥ 3 Curaçao criteria. In patient 1, we demonstrated that the acceptor splice site variant *ENG*(NM_001114753.3):c.68-2A > T causes an exon skipping that induces a frameshift with a premature stop codon. Transcripts carrying premature stop codons are unstable and would be degraded by the NMD mechanism, being consistent with previously published data that supports that HHT is caused by a loss of function mechanism [37,38]. The variant was initially classified as pathogenic according to the loss of function and the absence of the variant in population databases. The expertise of the clinical team in identifying specific HHT phenotypes allowed the activation of the PP4 criteria. In this case, cDNA analysis allowed the upgrade from only a prediction to a demonstrated abnormal splicing, and, therefore, loss-of-function was confirmed. New recommendations to differentiate between predictive versus functional evidence in the classification of splicing variants are currently being elaborated [39]. Not all variants located in the canonical acceptor and donor sites lead to an exon skipping since, depending on the strength of the splice site, some of them could induce intron retention [21]. This is the reason why RNA analysis is important when a novel splice site variant is identified.

In patient 2, the sequence variant *ENG*(NM_001114753.3):c.1311+4_1311+8del was initially classified as of uncertain significance. cDNA analysis showed that the variant induced an aberrant splicing due to intron retention. Therefore, the variant was reclassified from uncertain significance to likely pathogenic. The insertion of part of the intron in the coding sequence would lead to the insertion of 35 amino acids in the ZP domain involved in oligomerization and protein-protein interaction. Further studies would be needed to determine the precise effect of this variant on the protein functionality or even on the protein degradation if a defective folding is induced.

Finally, in patient 3, the acceptor splice site *ACVRL1*(NM_000020.3):c.526-6C > G was initially classified as of uncertain significance. cDNA analysis showed the skipping of one exon, introducing a frameshift variant with a premature stop codon that was predicted to be degraded by the activation of the nonsense-mediated mRNA decay mechanism. Therefore, the variant was reclassified as pathogenic. In this case, RNA analysis proved to be very useful for the assessment of variants located outside the canonical ± 1,2 splice sites, as it provides evidence to trigger loss of function criteria [39].

Splicing and frameshift variants are more common in the *ENG* gene (79 reported variants [20]) than in the *ACVRL1* gene (43 reported variants [19]) [40,41,42], being in line with our results. Interestingly, two variants affecting non-canonical splice sites (c.526-3C > G and c.526-7C > G) similar to the one found in patient 3 have been described in *ACVRL1*. The variant c.526-3C > G reported by Torring et al., (2014) [43] was identified in two affected family members that presented epistaxis, telangiectasia, and positive family history of HHT. No functional analysis of the variant was performed but it was predicted to have a pathogenic effect by three of the five splice site prediction tools used (Splicesitefinder-like [44], Maxentscan [45], Genesplicer [46], NNsplice [47] and Human Splicing Finder [48]). Argyriou et al., (2005) [49] reported the splice site acceptor variant c.526-7C > G that was predicted to skip the 3′ downstream exon. RNA analysis from peripheral blood using reverse transcription polymerase chain reaction (RT-PCR) with exonic primers that span the exon expected to be deleted, resulting in only the detection of the wild-type transcript. The affected individual presented epistaxis, telangiectasia, and positive family history of HHT. On the other hand, a canonical acceptor splice site variant located in the same region (c.526-1G > A) has been described in ClinVar (VCV000570721.8) and other databases [19,50]. The variant c.526-1G > A is predicted to abolish the acceptor splice site and disrupt RNA splicing, although no functional data is available. Additionally, Lesca et al., 2004 [40] and Bossler et al., 2006 [51] described a splice site acceptor variant c.68-1G > A in the *ENG* gene, similar to the one identified here (c.68-2A > T) in the two individuals with a clinical diagnosis of HHT.

In general, it is more common to upgrade variants of uncertain significance using PP1 (cosegregation criteria), following Jarvik and Browning criteria [52]. Unfortunately, although clinical interviews uncovered large families with a clinical diagnosis of HHT (meeting ≥ 3 Curaçao criteria) relatives (pedigrees in Supplementary Materials), it was not possible to perform genetic family testing to include >2 informative meiosis per patient. Affected family members were either deceased or lived abroad. This is a challenge when performing family segregation studies for adult-onset diseases, such as HHT. Compared to childhood-onset diseases, parental segregation studies may not be possible, and relatives could not share the same geographical area. Therefore, the PP1 criterion could not be included in the classification of these sequence variants. Consequently, RNA assays were the only way to power these variants, and, with the results here presented, strong evidence for pathogenicity was gathered. Upgrading variants of unknown significance is crucial, as the molecular diagnosis is the definite tool to confirm clinical suspicion when a specific phenotype is identified. However, in patients with rare diseases is not uncommon to have insufficient clinical criteria, and genetic testing is needed to establish a definite diagnosis that allows an accurate risk assessment for family members. It is especially important in HHT for those patients who meet less than three of the Curaçao criteria [53]. In fact, genetic testing has been found preferable to clinical Curaçao criteria amongst patients under 21 years old [54]. Describing new gene—rare disease associations is important to expand the possibilities of future genetic diagnostics avoiding unnecessary complementary tests. Here, we have characterized three new likely pathogenic/pathogenic splice site variants in three patients with a clinical diagnosis of HHT according to Curaçao criteria.

In conclusion, we support that RNA analysis is a useful tool to evaluate the effect of splice site variants in the clinical management of HHT patients. Achieving a genetic diagnosis is essential since it contributes to better clinical surveillance and family counseling. A multidisciplinary approach involving clinicians, genetic counselors, and geneticists is necessary when dealing with patients with rare diseases.

## Figures and Tables

**Table 1:** Clinical data of patients with splice site variants in *ENG* and *ACVRL1* genes.

ID	Sex	Age	Curaçao Criteria										Clinical Status		
			FH	Epistaxis	Telangiectasia				Visceral VMS				Min Hb (g/L)	CI (L/min/m^2^)	HHT Specific Treatment
					F	L	T	N	GI	Lu	Li	Br			
1	F	66	+	+	+	+	−	+	+	−	+	−	87	2.6	BVZ
2	F	74	+	+	+	+	−	+	n/a	−	−	n/a	118	3.78	Oral iron
3	F	54	+	+	+	−	+	+	+	−	+	−	140	2.76	Oral iron

+, presence; −, absence; n/a, non-assessed; FH, Family History; F, Fingers; L, Lips; T, Tongue; N, Nose; GI, Gastrointestinal; Lu, Lung; Li, Liver; Br, Brain; Hb, Hemoglobin; CI, Cardiac Index; BVZ, Bevacizumab.

## Data Availability

The data generated as part of this study is available on request to the corresponding author.

## Supplementary Materials

The following supporting information can be downloaded at: https://www.mdpi.com/article/10.3390/genes14030772/s1, Figure S1: Pedigree of the three HHT affected patients reported in this article. Table S1. Primers of *ENG* (NM_001114753.3) and *ACVRL1* (NM_000020.3) genes used for cDNA analysis.
