# Supplementary material for: Improving Hereditary Hemorrhagic Telangiectasia Molecular Diagnosis: A Referral Center Experience
Source: Genes (Basel). 2023 Mar 22;14(3):772. doi: 10.3390/genes14030772 (PMC10048779; doi:10.3390/genes14030772)
Supplement: Supplementary file 1 [file genes-14-00772-s001.zip › Figure S1 DEF.pdf]

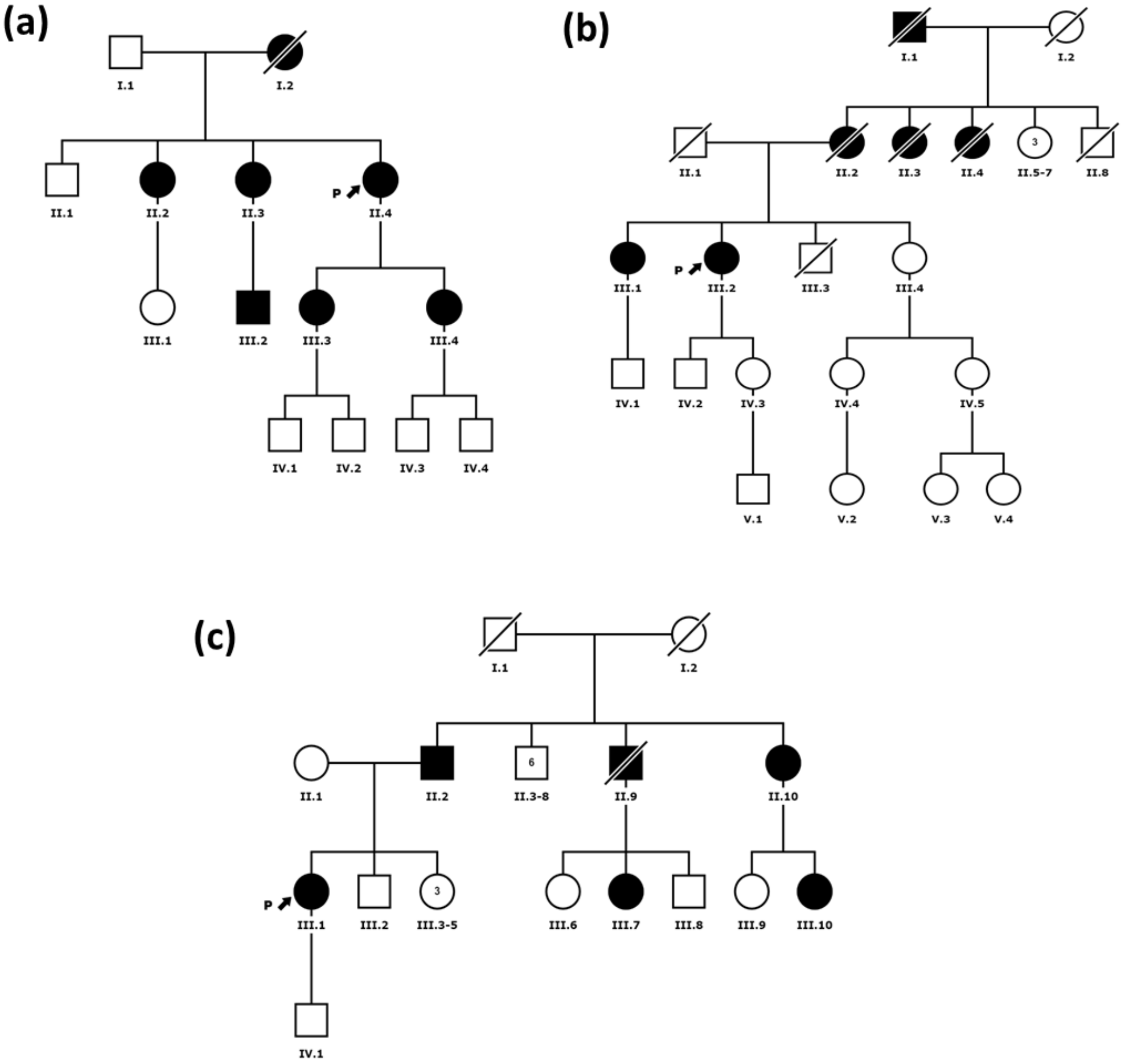

Figure S1. Pedigree of the three HHT affected patients carrying novel splicing variants reported. **(a)** Family tree of patient 1; **(b)** Family tree of patient 2 **(c)** Family tree of patient 3. Index patients are marked with an arrow, clinically affected HHT individuals are indicated by dark circles and squares.
