# Supplementary material for: Improving Hereditary Hemorrhagic Telangiectasia Molecular Diagnosis: A Referral Center Experience
Source: Genes (Basel). 2023 Mar 22;14(3):772. doi: 10.3390/genes14030772 (PMC10048779; doi:10.3390/genes14030772)
Supplement: Supplementary file 1 [file genes-14-00772-s001.zip › Table S1.pdf]

Table S1. Primers of *ENG* (NM_001114753.3) and *ACVRL1* (NM_000020.3) genes used for cDNA analysis.

Primer name	Primer sequence (5′→ 3′)
ENG 1-5Fw	**TGTAAAACGACGGCCAGT**GCAGCCCTGCCACTGGACAC
ENG 1-5Rev	**CAGGAAACAGCTATGACC**GAGCGTGCGGCCCATGTC
ENG 8-12Fw	**TGTAAAACGACGGCCAGT**CCTCCCAAGGACACTTGTAG
ENG 8-12Rev	**CAGGAAACAGCTATGACC**AGCAGGCTCACACAGTTGC
ACVRL1 4-7 Fw	**TGTAAAACGACGGCCAGT**GTCCTGGGCCTGTGGCATG
ACVRL1 4-7 Rev	**CAGGAAACAGCTATGACC**GTTCCCGGCTGCTCCGAAGG

The sequence in bold letters corresponds to M13.
